# Foetal disruptive brain injuries: Diagnosing the underlying pathogenetic mechanisms with cranial ultrasonography

**DOI:** 10.1111/dmcn.16380

**Published:** 2025-07-13

**Authors:** Ana Alarcón, Nuria Carreras, Tobias Muehlbacher, Dídac Casas‐Alba, Roberta Arena, Paola Roca‐Llabrés, Juan Navarro‐Morón, Linda S. de Vries, Paul Govaert, the EurUS.Brain group, Thais Agut, Roberta Arena, Ana Alarcón, Juan Arnáez, Marco Bartocci, Isabel Benavente‐Fernández, María Carmen Bravo, Fernando Cabañas, Nuria Carreras, Olivier Claris, Jeroen Dudink, Monica Fumagalli, Alfredo García‐Alix, Paul Govaert, Sandra Horsch, Simón Lubián, Tobias Muehlbacher, Alessandro Parodi, Adelina Pellicer, Luca Ramenghi, Charles C. Roehr, Simone Schwarz, Sylke Steggerda, Eva Valverde

**Affiliations:** ^1^ Department of Neonatology Hospital Sant Joan de Déu, BCNatal (Barcelona Centre for Maternal, Fetal and Neonatal Medicine) Barcelona Spain; ^2^ Department of Neonatology Hospital Clínic Barcelona, BCNatal (Barcelona Centre for Maternal, Fetal and Neonatal Medicine) Barcelona Spain; ^3^ Institut de Recerca Sant Joan de Déu Barcelona Spain; ^4^ Department of Surgery and Medical‐Surgical Specialties, Faculty of Medicine and Health Sciences Universitat de Barcelona Barcelona Spain; ^5^ Department of Neonatology University Hospital Zurich Zurich Switzerland; ^6^ Department of Genetics Hospital Sant Joan de Déu Barcelona Spain; ^7^ Department of Neonatology Ospedale Isola Tiberina‐Gemelli Isola Rome Italy; ^8^ Department of Paediatrics Hospital Germans Trias i Pujol Badalona Spain; ^9^ Department of Neonatology Hospital Universitario Costa del Sol Marbella Spain; ^10^ Department of Neonatology University Medical Center Utrecht Utrecht the Netherlands; ^11^ Department of Pediatrics, Division of Neonatology Leiden University Medical Center Leiden the Netherlands; ^12^ Department of Neonatology Brussels Belgium; ^13^ Department of Neonatology ZNA Middelheim Antwerp Belgium

## Abstract

Antenatal destructive events affecting the central nervous system of the foetus lead to disruptive brain lesions that are often associated with impaired neurodevelopment. The pathogenesis of these lesions encompasses a range of causes, including haemorrhagic, embolic, or other vascular events; exposure to teratogens, such as drugs or substance abuse; congenital brain infections; genetic conditions; and metabolic disorders. Cranial ultrasonography is the first‐line imaging modality to diagnose these antepartum brain lesions in the newborn infant; it is often complemented by brain magnetic resonance imaging to detect associated neuronal dysmigration and dysplasia. Using a pictorial approach, a differential diagnosis of foetal disruptive brain lesions and common findings related to antenatal brain damage can be made, including antenatal haemorrhagic and ischaemic brain injuries, porencephaly, schizencephaly, multicystic encephalomalacia, and hydranencephaly, as well as germinolytic cysts and lenticulostriate vasculopathy. The main conditions associated with foetal brain disruption, such as genetic cerebral vascular diseases, monochorionic twin pregnancies, congenital heart disease, maternal drug use, congenital infections, and inborn errors of metabolism can be used to illustrate typical imaging patterns that, when combined with clinical presentation, can assist in identifying the underlying mechanisms and causes, thus supporting individualized patient management.

## Graphical abstract

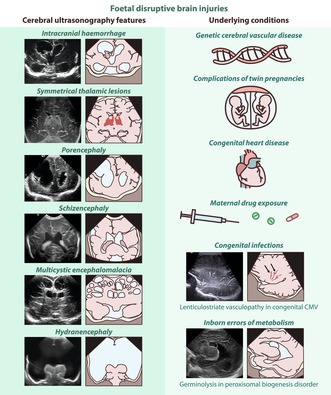

Plain language summary: https://onlinelibrary.wiley.com/doi/10.1111/dmcn.16431

**Abbreviations**
Chd: congenital heart disease
Cmv: cytomegalovirus
Csf: cerebrospinal fluid
Cus: cranial ultrasonography
Ich: intracranial haemorrhage
Iem: inborn error of metabolism
Ivh: intraventricular haemorrhage
Lsv: lenticulostriate vasculopathy
Sepc: subependymal pseudocyst

**What this paper adds**

- Teratogens, intrauterine infection, hypoxia‐ischaemia, haemorrhage, and trauma can cause disruptive brain injury in the foetus.
- This disruption can interfere with normal cellular organization and differentiation, leading to dysplastic changes.
- Disruptive foetal brain injuries are typically sporadic, but some neuroimaging patterns or familial presentation may indicate an underlying genetic disorder.
- Integrated neuroimaging findings, clinical manifestations, and genetic testing enhance aetiological diagnosis, and individualized treatment and management strategies.

Disruptive brain abnormalities result from a destructive process affecting the foetal central nervous system (CNS).^1^ These destructive processes can disturb typical brain development, often leading to associated developmental disorders, including abnormal cell proliferation, migration, and organization. They may arise from teratogens (including certain medications, illicit drugs, and infectious pathogens) and from vascular or mechanical mechanisms of tissue compromise. Consequently, most cases are sporadic; however, an underlying genetic disorder may sometimes be present, and it is often associated with an increased risk of vascular disruption.

This review covers antenatal haemorrhagic and ischaemic brain injuries, and encephaloclastic anomalies, such as porencephaly, schizencephaly, antenatal multicystic encephalomalacia, and hydranencephaly. It also addresses germinolytic cysts and lenticulostriate vasculopathy (LSV), common neonatal cranial ultrasonography (CUS) findings that are not intrinsically pathological but may be associated with congenital infections or other teratogenic entities. Additionally, we examine the conditions that increase the risk of foetal disruptive brain injuries, including monochorionic twin pregnancies, congenital heart disease (CHD), maternal drugs, congenital infections, and inborn errors of metabolism (IEMs). We also review genetic disorders that predispose to foetal stroke or antenatal brain disruption.

Our aim is to enhance diagnosis using neonatal neuroimaging, with a particular focus on CUS because most patterns of foetal disruptive brain disease have been well categorized using this technique.^2^ Our review is intended to help clinicians identify recognizable imaging phenotypes that, when considered alongside clinical presentation, may support an aetiological diagnosis, ultimately facilitating parent counselling, informed treatment decisions, and planning of individualized follow‐up and early intervention programmes.

## ANTENATAL INTRACRANIAL HAEMORRHAGE

**FIGURE 1:**
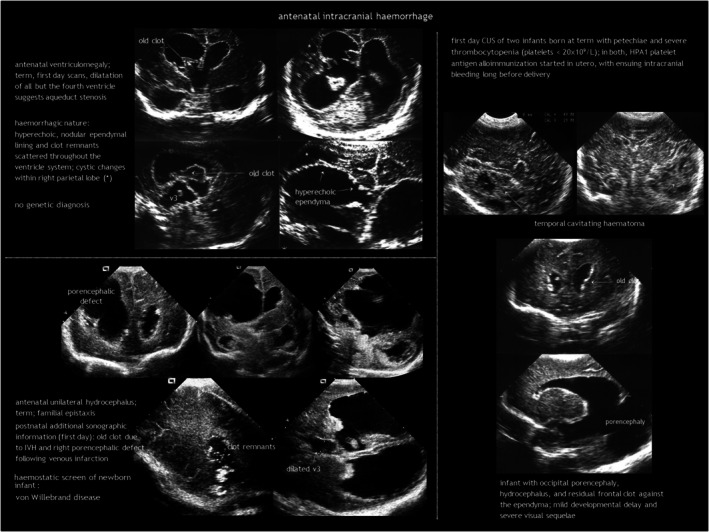
Antenatal intracranial haemorrhage. Examples of unexplained and explained cases. Abbreviations: CUS, cranial ultrasonography; HPA1, human platelet antigen 1; IVH, intraventricular haemorrhage.

While intracranial haemorrhage (ICH) is relatively common in infants born preterm, foetal ICH is rare, estimated at 0.1 to 1 per 1000 pregnancies,^3^ or up to 1% to 3% when using foetal magnetic resonance imaging (MRI).^4^, ^5^ Intraventricular haemorrhage (IVH) is the most common ICH in the foetus^2^, ^3^ (Figure 1). Cerebellar haemorrhage may be isolated or concurrent with supratentorial IVH.^6^ The causes of antenatal ICH are often elusive but can include bleeding diathesis, genetic abnormalities, infection, trauma, tumours, and vascular malformations.^7^ Twin‐to‐twin transfusion syndrome and intrauterine blood transfusion for foetal anaemia can also be complicated by ICH.^8^

Diagnosis of foetal ICH involves antenatal imaging or CUS immediately after birth. IVH usually originates from fragile veins in the germinal matrix, typically in the caudothalamic notch. Recent haemorrhages are hyperechoic. In the following days, they retract and show internal cavitation. By the end of the first week, a sterile inflammatory response transforms the ventricle lining into a distinct thick line. Static or progressive ventricular dilatation may follow. Post‐haemorrhagic hydrocephalus is typically communicating, resulting from impaired cerebrospinal fluid (CSF) resorption. Less commonly, it can be non‐communicating because of ventricular obstruction from a clot or scarring. However, after the acute event, the primary mechanism is obstruction at the basal cisterns and obliterative arachnoiditis caused by blood and the associated inflammatory response. IVH can be accompanied by periventricular haemorrhagic infarction, ultimately resulting in the formation of a porencephalic cyst.

In utero cerebellar haemorrhage is hyperechoic on antenatal CUS in the acute phase, becoming hypoechoic in the subacute phase. It may disrupt cerebellar development, leading to (asymmetrical) cerebellar hypoplasia and dysplasia.^8^, ^9^

Unlike postnatal ICH, foetal ICH is typically diagnosed after its onset, which hinders early assessment and grading of the acute haemorrhage.^3^ Late findings may include clot remnants, ventriculomegaly with irregular borders, or porencephaly. Susceptibility‐weighted imaging and gradient echo MRI can reveal haemosiderin staining long after the acute phase.

The aetiological work‐up (similar to postnatal ICH) includes investigating drug exposure, trauma, TORCH infections, isoimmune and alloimmune thrombocytopenia, coagulation disorders, sinovenous thrombosis, and vascular malformations (Table 1). However, a plausible cause is identified in a minority of cases.^3^ The bleeding pattern may suggest the aetiology; for example, alloimmune thrombocytopenia typically presents with superficial parenchymal haemorrhage in the temporal lobe.^10^ Genetic testing in foetal ICH can identify variants in genes related to haemostatic or prothrombotic disorders, and genes such as *COL4A1* and *COL4A2*, *JAM3*, *GATA1*, and *USP18*.^11^, ^12^, ^13^, ^14^ Besides monogenic disorders, combinations of genetic polymorphisms may contribute to the risk of ICH. Genetic analysis can potentially facilitate individualization of treatment, genetic counselling, and primary and secondary prevention of ICH.

**TABLE 1:** Recommended aetiological testing in foetal disruptive brain abnormalities^a^.

Type of injury	Aetiological investigations
Intracranial haemorrhage	Maternal history: drug use, trauma
	Testing for TORCH infections
	Platelet count, antiplatelet antibodies, clotting
	Haemorrhage pattern on imaging: intraventricular, intraparenchymal, venous infarction (including sinovenous thrombosis), haemorrhagic transformation of arterial infarction, tumour‐related, vascular malformation
	Consider genetic testing if atypical presentation: monogenic disorders (*COL4A1* and *COL4A2*, *JAM3*, *GATA1*, *USP18* variants), polymorphisms in thrombophilia‐associated genes
Ischaemic stroke	Maternal history: hypotension, trauma, or drug use (e.g. cocaine, tobacco)
	Obstetric factors: twin‐to‐twin transfusion syndrome, co‐twin death, umbilical cord abnormalities, placental insufficiency
	Testing for TORCH infections
	Consider PHACE syndrome, *COL4A1* and *COL4A2* variants, and thrombophilia work‐up
Symmetrical thalamic injury	Maternal history: hypotension, trauma, or drug use
	Umbilical cord or placental events
	History of severe carbon monoxide exposure
	Investigate inborn errors of metabolism (mitochondrial disorders, pyruvate metabolism disorders, molybdenum cofactor deficiency)
	Test for TORCH infections
Ischaemic injury to dorsal brainstem with Möbius sequence	Maternal factors: misoprostol use to induce abortion in the first trimester, cocaine abuse
	Foetal vascular events: thrombus, embolism, haemorrhage
Porencephaly and schizencephaly	Same as for arterial ischaemic stroke or periventricular haemorrhagic infarction, depending on the pattern; consider *COL4A1* and *COL4A2* variants
Multicystic encephalomalacia	Maternal history: hypotension, trauma, or drug use
	Obstetric factors: twin‐to‐twin transfusion syndrome, co‐twin death, umbilical cord or placental events
	TORCH screening
	Inborn errors of metabolism (pyruvate dehydrogenase deficiency, molybdenum cofactor deficiency)
Hydranencephaly	Maternal hypotension or hypoxia, teratogen exposure, co‐twin death, extensive haemorrhagic venous infarction
	TORCH infections
	Genetic testing: *COL4A1* and *COL4A2*, *FLVCR2* (Fowler syndrome), *CEP55* (MARCH syndrome)
Subependymal pseudocysts	Aetiological investigation according to clinical suspicion (Table 3)
Lenticulostriate vasculopathy	Aetiological investigation according to clinical suspicion (Table 4)

^a^ Although aetiological investigation is advised, most of these conditions are sporadic and most often idiopathic. Abbreviations: MARCH, multinucleated neurons, anhydramnios, renal dysplasia, cerebellar hypoplasia, and hydranencephaly; PHACE, posterior fossa abnormalities, haemangiomas, arterial lesions, cardiac abnormalities, and eye problems; TORCH, toxoplasmosis, other agents (classically referring to syphilis), rubella, CMV, and herpes simplex virus.

Antenatal ICH has a variable outcome, according to its extent and associated parenchymal injury.^3^, ^15^, ^16^ In mild cases, resorption and resolution without sequelae can occur. However, mortality ranges from 15% to 30%.^15^, ^16^ Shunt placement is required in one‐third of cases and in 50% of those with post‐haemorrhagic ventricular dilatation.^15^, ^16^ Periventricular haemorrhagic infarction is associated with a higher risk of mortality, motor impairment (if the corticospinal tract is involved), epilepsy, and cognitive deficits.^3^, ^15^

## ANTENATAL ISCHAEMIC BRAIN INJURY

### Foetal ischaemic stroke and global forebrain ischaemia

Both arterial or venous ischaemic stroke and global forebrain ischaemia can occur in utero, resulting from systemic hypotension (e.g. maternal hypotension and cardiac arrest, trauma, twin‐to‐twin transfusion syndrome, and umbilical cord or placental events); vascular constriction (e.g. posterior fossa abnormalities, haemangiomas, arterial lesions, cardiac abnormalities, and eye problems [PHACE] syndrome), vasospasm from maternal cocaine abuse, vessel wall disruption in *COL4A1* and *COL4A2* disorders; or vascular occlusion by an embolus, thrombus, or vasculitis (e.g. co‐twin death, placental thrombosis, TORCH infections, or hypercoagulability) (Table 1). Ischaemic brain injury results in cell death and gliosis or cavitation, particularly in areas with complete infarction. The injured immature brain is prone to cavitation because of its high water content, limited myelinated fibre tracts, and restricted astroglial response.

**FIGURE 2:**
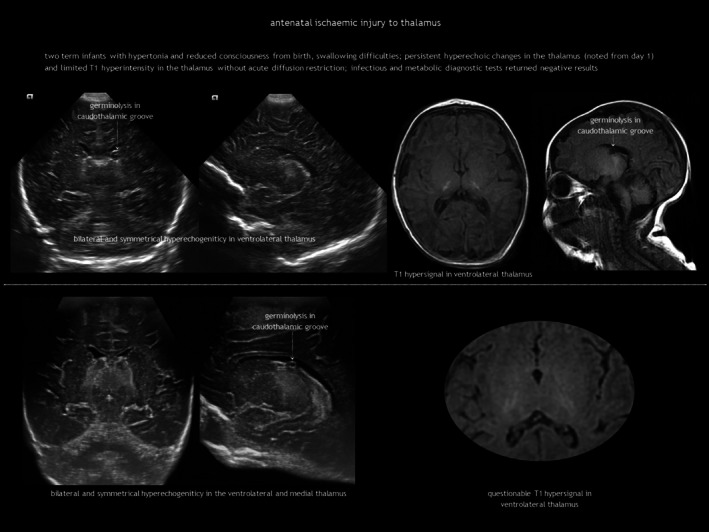
Antenatal symmetrical thalamic lesions.

In antenatal global forebrain injury, surviving acute near‐total asphyxia results in established brain damage, primarily affecting the thalami, hippocampi, and dorsal brainstem.^17^, ^18^ Symmetrical thalamic lesions, combined with a consistent clinical presentation, can suggest a tentative diagnosis (Figure 2). Clinical features include those of foetal hypokinesia sequence (polyhydramnios, pulmonary hypoplasia, joint contractures). Neurological examination at birth reveals hypertonia, hyperreflexia, facial diplegia, and dysphagia. Prognosis is generally poor. Limited CUS descriptions report bilateral thalamic increased echogenicity because of gliosis and mineralization, often with subtle signs of atrophy.^18^ Changes on MRI may be even more subtle. Diagnosis is ultimately one of exclusion, requiring the ruling out of causes such as primary haemorrhage, foetal infection, and IEMs, including mitochondrial and pyruvate metabolism disorders, as well as molybdenum cofactor deficiency (Table 1). Involvement of the globus pallidus may indicate a genetic–metabolic disease or severe carbon monoxide exposure.^19^, ^20^

**FIGURE 3:**
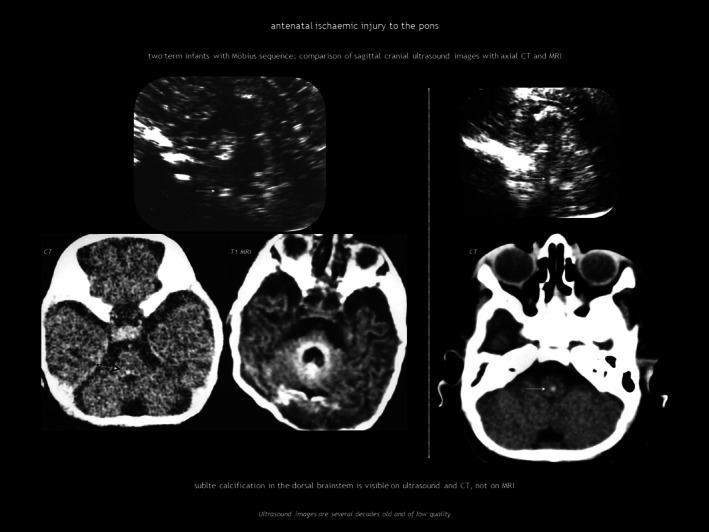
Antenatal ischaemic injury to the pons. Abbreviations: CT, computed tomography; MRI magnetic resonance imaging.

Ischaemic injury of the pons during embryogenesis can cause Möbius sequence, a rare disorder characterized by congenital facial palsy and external ophthalmoplegia because of involvement of the motor nuclei of cranial nerves VI and VII. CUS and computed tomography (CT) show calcification in the dorsal brainstem immediately anterior to the fourth ventricle (Figure 3).^21^, ^22^

### Congenital porencephaly and schizencephaly

**FIGURE 4:**
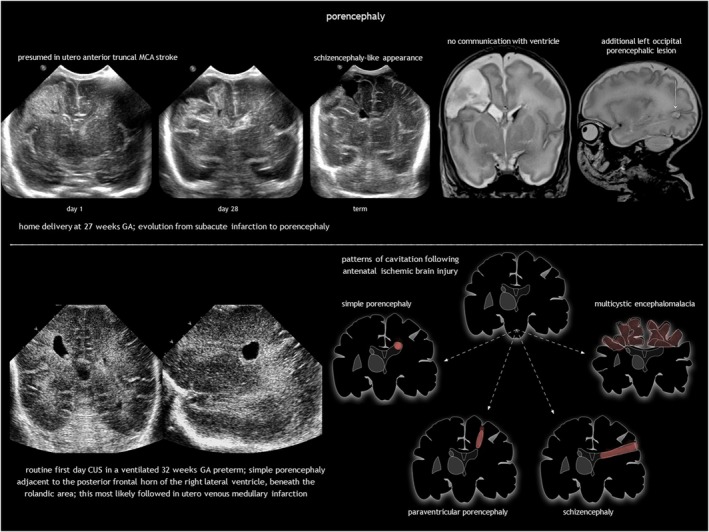
Porencephaly. Abbreviations: CUS, cranial ultrasonography; MCA, middle cerebral artery.

**FIGURE 5:**
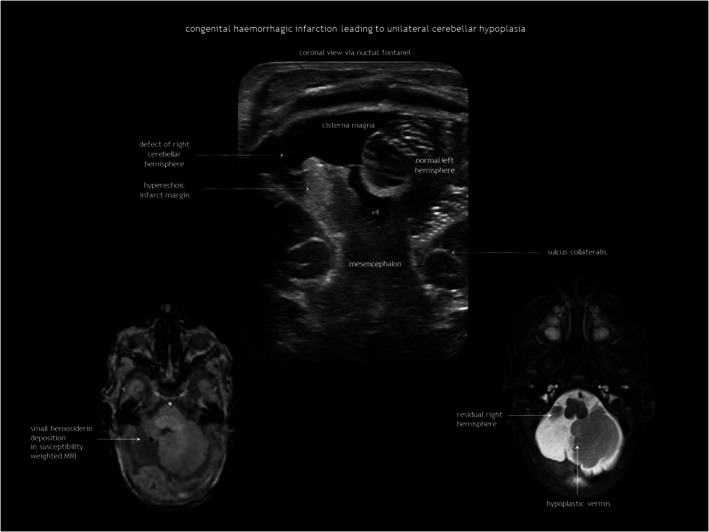
Unilateral cerebellar hypoplasia. Infant born preterm at 31 weeks of gestation with antenatal haemorrhagic disruption of the cerebellum leading to severe hypoplasia of the right cerebellar hemisphere and inferior vermis. Abbreviation: MRI, magnetic resonance imaging.

Antenatal brain necrosis and tissue destruction can result from haemorrhage, ischaemia, infection, or trauma. Congenital porencephalic cysts (Figure 4), caused by third‐trimester injury, are fluid‐filled cavities lined with white matter. Second‐trimester vascular damage can cause cortical dysplasia (polymicrogyria or heterotopia), with or without a parenchymal cleft, going from the subarachnoid space to the lateral ventricle (schizencephaly). Although rare, focal arterial infarction of the cerebellum in utero may lead to cerebellar dysplasia (Figure 5). Porencephaly and schizencephaly are usually sporadic (Table 1), although some cases have been associated with *COL4A1*‐related and *COL4A2*‐related disorders.^23^

Porencephalic cysts are typically connected to the ventricular system, the subarachnoid space, or both. Arterial ischaemic stroke leads to porencephaly across the brain mantle, while periventricular haemorrhagic infarction causes deep white matter porencephaly. Arachnoid cysts (sharply defined extracerebral cysts potentially causing mass effect) and rare tumours with mixed fluid and solid components (teratoma, pilocytic astrocytoma) should be considered in the differential diagnosis. Prognosis for porencephaly depends on size and location (and specifically involvement of the corticospinal tract).

**FIGURE 6:**
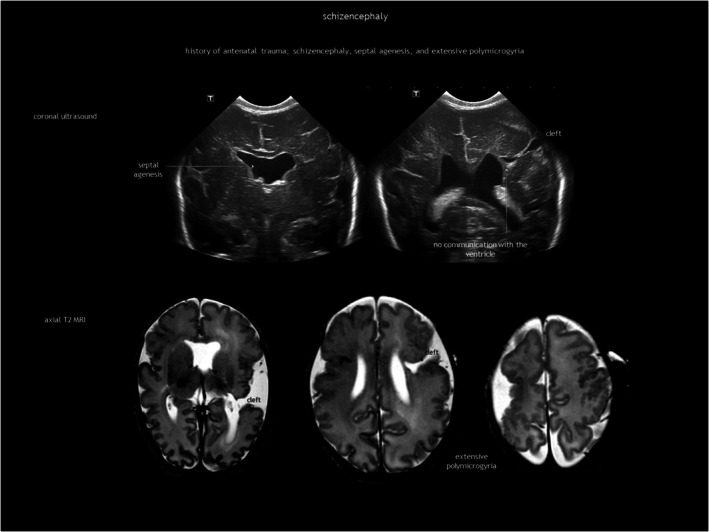
Type 3 schizencephaly. Abbreviation: MRI, magnetic resonance imaging.

**FIGURE 7:**
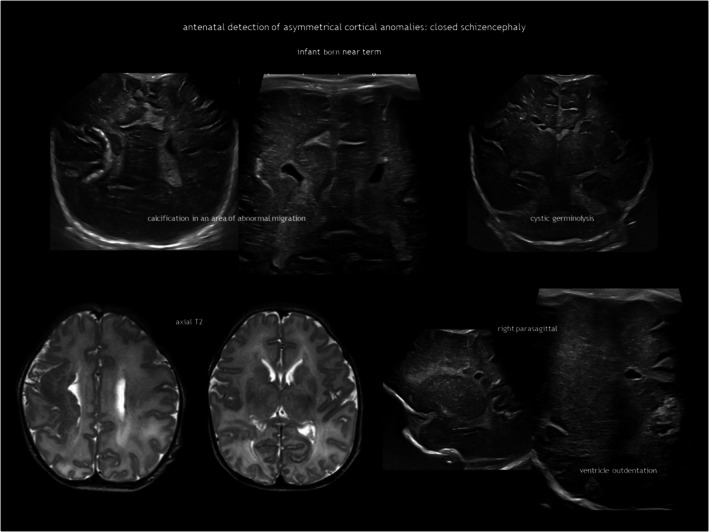
Type 1 schizencephaly.

Schizencephaly is a CSF‐containing cleft running between the pial and the ependymal surfaces (Figure 6). A broader definition also includes a transmantle column of dysplastic grey matter extending from the ependyma to the pia without a CSF cleft^24^ (Figure 7). Griffiths recently classified schizencephaly into type 1 (transmantle column of abnormal grey matter but no evidence of a CSF‐containing cleft, called ‘transmantle heterotopia’ by other authors), type 2 (CSF‐containing cleft lined with abutting lips of abnormal grey matter, traditionally known as ‘closed‐lip’ schizencephaly), and type 3 (CSF‐containing cleft lined with non‐abutting lips of abnormal grey matter, also known as ‘open‐lip’ schizencephaly).^25^ Schizencephaly has a very low prevalence of approximately 1.5 per 100 000 liveborn infants, with less than half of cases detected antenatally.^26^ Location is predominantly frontal and parietal. There may be more than one area in the same hemisphere, and bilateral schizencephaly is reported in 40% to 50% of cases.^25^, ^27^ Schizencephaly is associated with absent or disrupted septum pellucidum in approximately 70% of cases.^25^ Regarding prognosis, children with a unilateral closed cleft may present with epilepsy or spastic unilateral cerebral palsy if the corticospinal tract is involved. In addition to often refractory epilepsy, bilateral open clefts can present as microcephaly, spastic multilateral palsy, and cognitive impairment.

### Antenatal multicystic encephalomalacia and hydranencephaly

**FIGURE 8:**
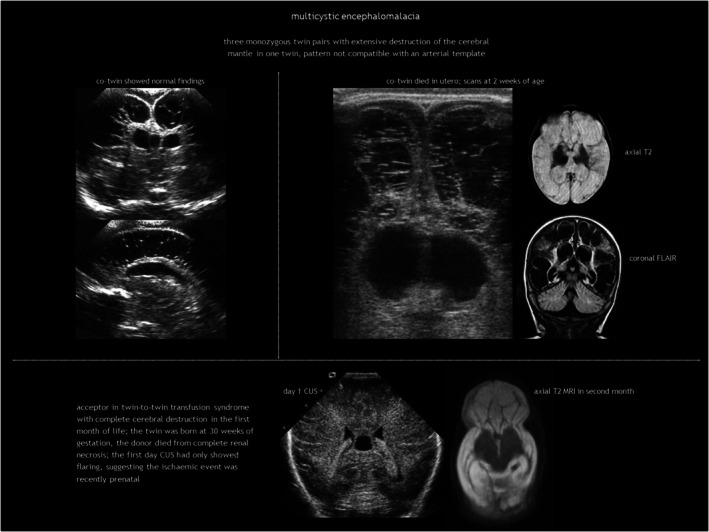
Multicystic encephalomalacia. Abbreviations: CUS, cranial ultrasonography; FLAIR, fluid‐attenuated inversion recovery; MRI magnetic resonance imaging.

Antenatal multicystic encephalomalacia is the result of third‐trimester prolonged, partial hypoxic‐ischaemic injury (Table 1). It consists of bilateral, diffuse multiloculated white matter cavitation, which ensues weeks after the insult (Figure 8). It typically affects the arterial border zones. There is no communication between the cysts and the ventricular system. The cortex is ulegyric (shrunken and flattened) and the CSF spaces are enlarged because of brain volume loss, with thinning of the corpus callosum.

**FIGURE 9:**
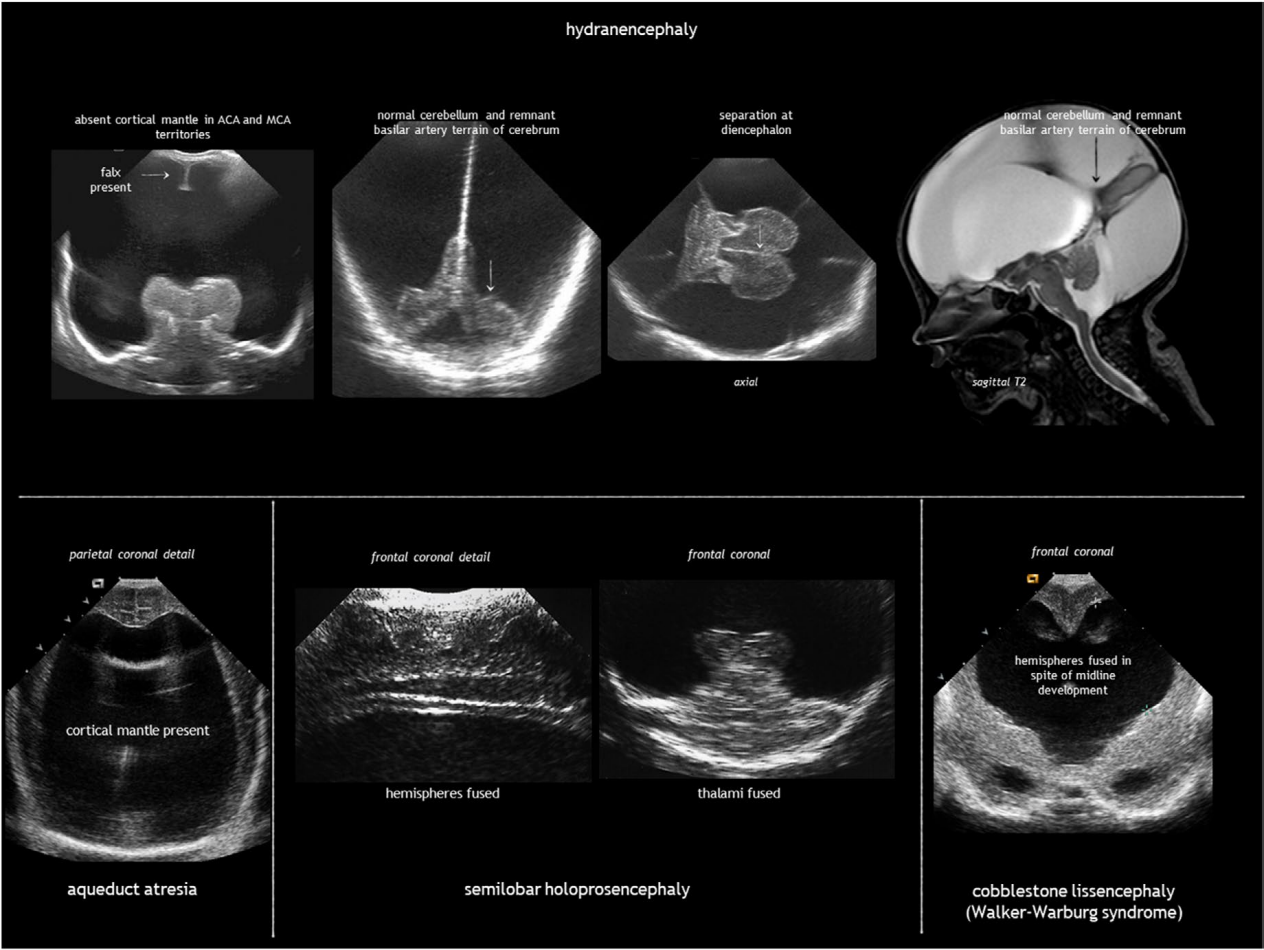
Hydranencephaly and its differential diagnosis on cranial ultrasound. Abbreviations: ACA, anterior cerebral artery; MCA, middle cerebral artery.

Hydranencephaly is an infrequent condition seen in 1 to 2 of 10 000 pregnancies and in 1.4 to 2.8 per 100 000 live births.^28^, ^29^ It is characterized by destruction of both cerebral hemispheres, which are replaced with a gliomeningeal sac filled with CSF (Figure 9). The skull is present. The most accepted pathogenesis is massive bilateral supraclinoid internal carotid artery occlusion, with an onset as early as 8 to 12 weeks of gestation.^30^ As the posterior cerebral arteries and basilary arteries are typically not involved, the basal ganglia, brainstem, and posterior fossa are spared. When vascular disruption affects the middle cerebral arteries instead of the internal carotid arteries, the hippocampus, basal parts of the temporal lobes, occipital lobes, and orbitofrontal cortex are preserved. Hydranencephaly is nearly always associated with hydrocephalus, most probably because of aqueduct stenosis. Otherwise, the condition is called microhydranencephaly.

Although the aetiology is often elusive, potential causes include forebrain hypoperfusion or stroke (because of maternal hypotension or hypoxia; teratogens such as cocaine, smoking, oestrogens, or sodium valproate; or co‐twin death), vasculopathy secondary to congenital infection, and extensive haemorrhagic venous infarction (Table 1). Syndromic or monogenic causes of hydranencephaly are rare, but pathogenic variants in *COL4A1* have been linked to the condition via vessel wall disruption.^31^ A homozygous *LAMB1* variant has been reported in a single case of hydranencephaly.^32^ However, additional clinical and functional evidence is needed to establish a causal relationship.

Fowler syndrome, also known as proliferative vasculopathy and hydranencephaly‐hydrocephaly syndrome, is a rare autosomal recessive disorder characterized by hydranencephaly‐hydrocephaly, a foetal akinesia sequence, and glomerular vasculopathy in the CNS and retina, along with diffuse ischaemic lesions with dystrophic calcification in the brainstem, basal ganglia, cerebellum, and spinal cord.^33^, ^34^ It is caused by biallelic pathogenic variants in *FLVCR2*. Hydranencephaly has also been reported in ‘multinucleated neurons, anhydramnios, renal dysplasia, cerebellar hypoplasia, and hydranencephaly’ (MARCH) syndrome associated with biallelic *CEP55* pathogenic variants.^35^, ^36^ In this disorder, the abnormality results from arrested neuronal mitosis rather than a vascular mechanism.

Most patients with hydranencephaly die in utero. Infants born alive can breathe, suck, and swallow because of brainstem integrity. Survivors develop spastic cerebral palsy, severe developmental delay, epilepsy, and visual impairment. Most die in the first year, although a few survive to adulthood.^30^ Treatment involves supportive management of symptoms and complications. Ventriculoperitoneal shunt placement may palliate progressive hydrocephalus but does not improve prognosis. Choroid plexus coagulation and choroid plexectomy are also proposed for treating hydranencephaly or extreme hydrocephalus.^37^, ^38^

The main differential diagnoses for hydranencephaly are extreme hydrocephalus and alobar holoprosencephaly. Unlike hydranencephaly, in massive hydrocephalus there is a thin rim of cerebral mantle. Severe hydrocephalus may destroy the septum pellucidum but the midline structures (falx, interhemispheric fissure, and corpus callosum) can still be identified. Alobar holoprosencephaly is often associated with midline facial abnormalities; neuroimaging shows a large central monoventricle with absent falx, interhemispheric fissure, corpus callosum, optic tracts, olfactory bulbs, and septum pellucidum; the central grey nuclei are fused in the midline.

## GENETIC CAUSES OF ANTENATAL STROKE

Perinatal stroke occurs between 20 weeks of gestation and 28 days after birth.^39^ It includes a heterogeneous group of disorders, classified as ischaemic (arterial or venous) or haemorrhagic, and further characterized by the timing of presentation (foetal, neonatal, or presumed perinatal). Although paediatric and perinatal stroke is mostly sporadic, an increasing number of associated monogenic disorders have been described (Table 2).^14^, ^23^, ^31^, ^40^, ^41^, ^42^, ^43^, ^44^, ^45^, ^46^, ^47^, ^48^, ^49^, ^50^, ^51^, ^52^, ^53^, ^54^, ^55^, ^56^, ^57^, ^58^, ^59^, ^60^, ^61^, ^62^, ^63^, ^64^, ^65^, ^66^, ^67^ A detailed description of each condition is beyond the scope of this review.

**TABLE 2:** Main genetic and syndromic disorders associated with paediatric stroke.

Disease and associated gene(s)	Stroke pathogenesis
*COL4A1*‐related and *COL4A2*‐related disorders^14^, ^23^, ^31^, ^40^, ^42^, ^43^, ^44^, ^45^, ^46^, ^47^	Basement membrane disorder
*ACTA2*‐related vasculopathy^40^, ^48^	Smooth muscle dysfunction
Marfan syndrome (*FBN1*)^40^, ^49^ Ehlers–Danlos syndrome type IV (*COL3A1*)^40^, ^41^ Ehlers–Danlos syndrome type IVA (*PLOD1*)^40^, ^50^ Loeys–Dietz syndrome (*TGFBR1*)^51^	Connective tissue disorders
PHACE syndrome^40^, ^41^, ^52^	Sporadic, unknown aetiology. Cervical and intracranial arterial anomalies
Menkes disease (*ATP7A*)^40^, ^53^	Copper metabolism disorder, vascular tortuosity
Hutchinson–Gilford progeria (*LMNA*)^40^, ^41^, ^54^	Abnormal progerin production in cells, essential for vascular structure and function
Aicardi–Goutières syndrome (several genes),^40^, ^55^, ^56^, ^57^, ^58^ USP18 deficiency,^13^, ^56^ and others	Type I interferonopathies causing inflammatory vasculopathy
Incontinentia pigmenti (*IKBKG*)^59^	X‐linked. NF‐κB essential modulator. Obliteration of small and medium‐sized arteries
Haemorrhagic destruction of the brain, subependymal calcification, and cataracts (*JAM3*)^12^, ^55^, ^56^	Tight‐junction dysfunction
Von Hippel–Lindau syndrome (*VHL*)^40^, ^41^, ^60^	CNS haemangioblastoma, risk of haemorrhagic stroke
Sturge–Weber syndrome (*GNAQ*)^40^, ^41^, ^61^	Vascular malformation; risk of thrombosis, haemorrhage, and stroke‐like episodes
Neurofibromatosis type 1 (*NF1*)^40^, ^41^, ^62^	Moyamoya arteriopathy, cerebral aneurysms, stenotic or ectatic cerebral vessels; risk of ischaemic or haemorrhagic complications
Capillary malformation‐arteriovenous malformation syndrome (*RASA1* and *EPHB4*)^63^ Hereditary haemorrhagic telangiectasia (several genes)^64^	Arteriovenous malformation, risk of ischaemic or haemorrhagic complications
Fabry disease (*GLA*)^40^, ^41^, ^65^	Accumulation of globotriaosylceramide (GL‐3) within lysosomes in different cell types, including endothelial cells
Mitochondrial disorders (several genes)^41^, ^66^ Molybdenum cofactor deficiency (several genes)^67^	Metabolic stroke (acute cellular energy failure)

Abbreviations: CNS, central nervous system; PHACE, posterior fossa abnormalities, haemangiomas, arterial lesions, cardiac abnormalities, and eye problems; USP18, ubiquitin specific peptidase 18.

**FIGURE 10:**
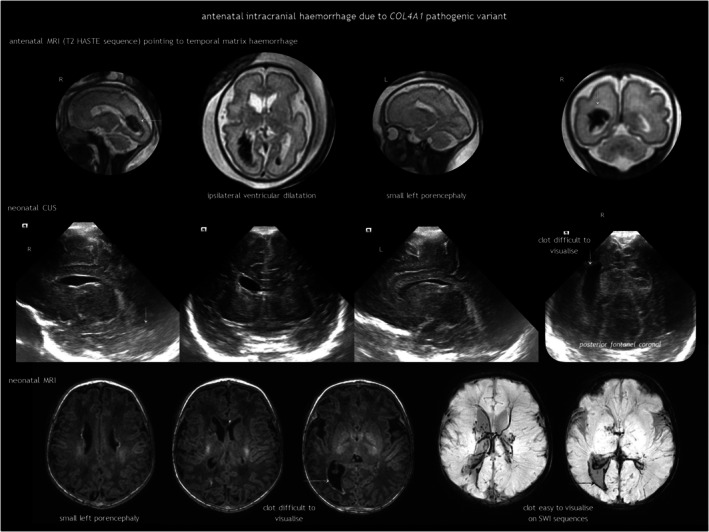
Antenatal intracranial haemorrhage associated with a *COL4A1* pathogenic variant. At 27 weeks of gestation, intraventricular haemorrhage and periventricular haemorrhagic infarction were detected. Magnetic resonance imaging (MRI) at 30 weeks confirmed these findings; a second MRI at 33 weeks showed porencephaly. A de novo *COL4A1* c.4652G>A (p.Cys1551Tyr) variant was identified in heterozygosity. The infant was born at 39 weeks. Abbreviations: CUS, cranial ultrasonography; MRI magnetic resonance imaging; SWI, susceptibility‐weighted imaging.

However, *COL4A1*‐related and *COL4A2*‐related disorders deserve special attention. What was previously known as familial porencephaly is now recognized as being caused by heterozygous pathogenic variants in *COL4A1* and *COL4A2*. These variants are associated with a broad spectrum of cerebrovascular diseases, with an onset from foetal life onwards and variable severity, including small‐vessel disease, leukoencephalopathy, and extensive intraparenchymal haemorrhage.^42^, ^43^, ^44^, ^45^, ^46^, ^47^ Although not common, recurrent ICH has been reported.^43^ No clear genotype–phenotype correlation has been established to date.^45^ Regarding foetal brain disruption, *COL4A1* and *COL4A2* pathogenic variants are a known cause of congenital porencephaly (Figure 10) and transmantle lesions, including schizencephaly and hydranencephaly.^23^, ^31^ Clinical presentation ranges from severe neurological condition to mild or asymptomatic, with epilepsy as the predominant feature.^45^ *COL4A1* and *COL4A2* pathogenic variants cause multisystem disease. Extraneural involvement includes ocular abnormalities (such as congenital cataract, retinal vessel tortuosity, and anterior chamber dysgenesis), increased serum creatine kinase or muscle cramps, renal abnormalities (haematuria, hydronephrosis, renal agenesis, and polycystic kidneys), and cardiac disease.^45^ There is no treatment for *COL4A1*‐related and *COL4A2*‐related disorders. Prevention focuses on avoiding head trauma and considering anticoagulation and thrombolysis when appropriate.

Apart from the single‐gene disorders listed in Table 2, the risk of paediatric and perinatal stroke or its recurrence may be influenced by polymorphisms in thrombophilia‐associated genes, including those coding for methylenetetrahydrofolate reductase, factor V Leiden, prothrombin, factor XIII, plasminogen activator inhibitor, human platelet antigens, and apolipoprotein E.^68^, ^69^, ^70^ While single polymorphisms may not be clinically significant, a cumulative effect of prothrombotic risk factors may influence perinatal and paediatric stroke. Identifying these risk factors is valuable for potential primary and secondary prevention.

The yield of genetic studies in perinatal cerebrovascular disruption is low; testing could be restricted to patients with complex syndromic phenotypes suggestive of a monogenic disorder, a family history of antenatal or early‐onset cerebrovascular disease, or recurrent stroke.

## GERMINOLYSIS AND VENTRICULAR ADHESIONS

**FIGURE 11:**
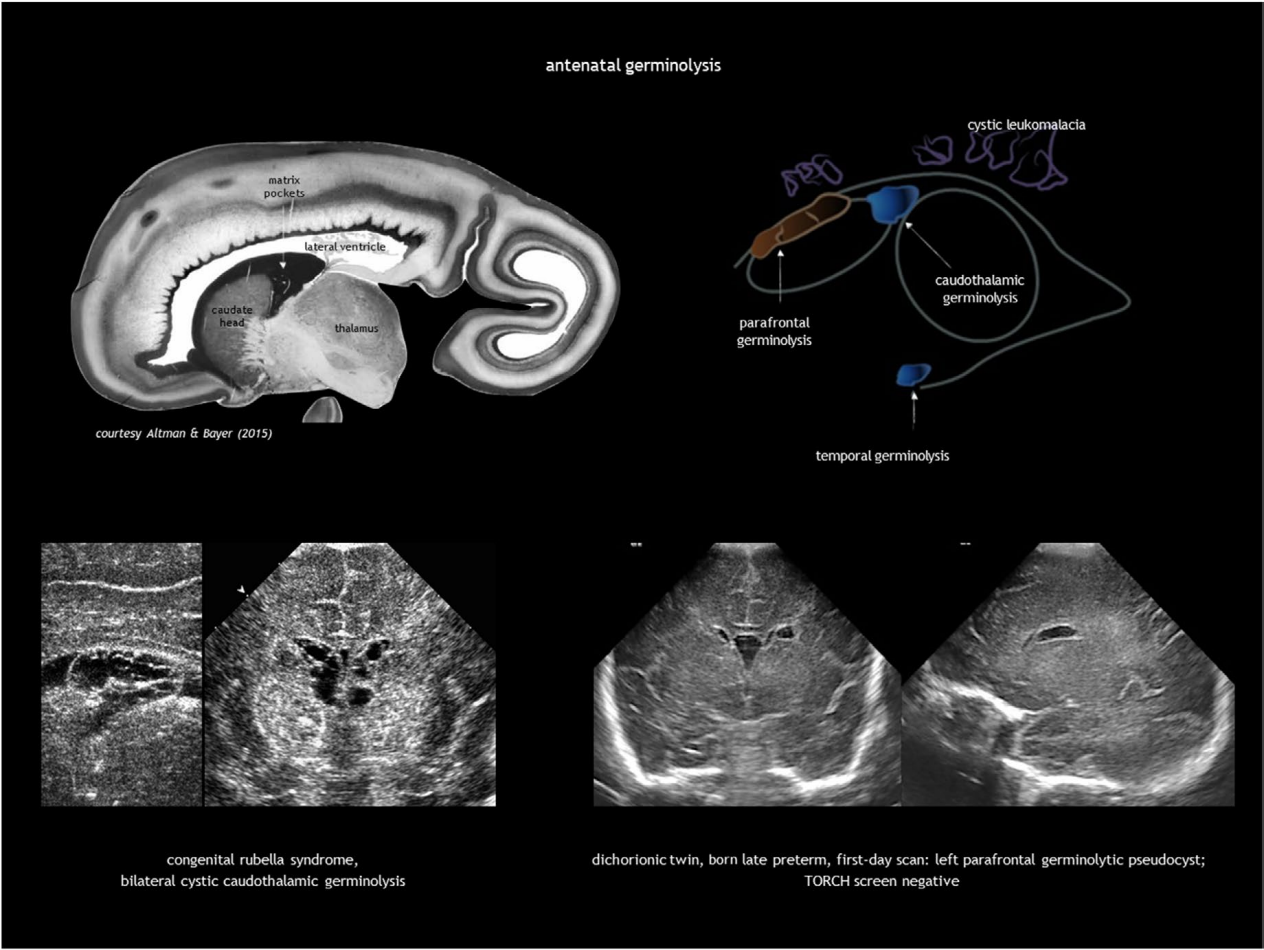
Antenatal germinolysis. Abbreviation: TORCH, toxoplasmosis, other agents (classically referring to syphilis), rubella, cytomegalovirus, and herpes simplex virus.

Subependymal pseudocysts (SEPCs) result from germinolysis (germinal matrix cystic regression probably after haemorrhage or microinfarction).^71^ The term pseudocyst indicates the absence of an ependymal lining. They should not be called ‘periventricular cysts’ because this may incorrectly imply periventricular leukomalacia. The most common location for SEPCs is the caudothalamic notch, while parafrontal and temporal germinolyses is less frequent (Figure 11). As the germinal matrix recedes around 28 weeks of gestation, caudothalamic pockets persist until 34 to 35 weeks, making frontal and temporal germinolyses likely to occur earlier. SEPCs are often bilateral but not necessarily symmetrical.

Caudothalamic SEPCs can be single or multiple, appearing as multilocular rounded prominences protruding into the lateral ventricle. Caudothalamic germinolysis is well illustrated using CUS, while MRI may not detect small cysts a few millimetres in diameter.

In parafrontal germinolysis, SEPCs are adjacent to the lateral walls of the frontal horns of the lateral ventricles. They are positioned more medially and lower than the cysts of periventricular leukomalacia, and more laterally and anteriorly than caudothalamic germinolytic SEPCs. In the coronal plane, they may appear as enlarged frontal horns, while in the parasagittal section, they are elongated and sometimes contain septa.

**FIGURE 12:**
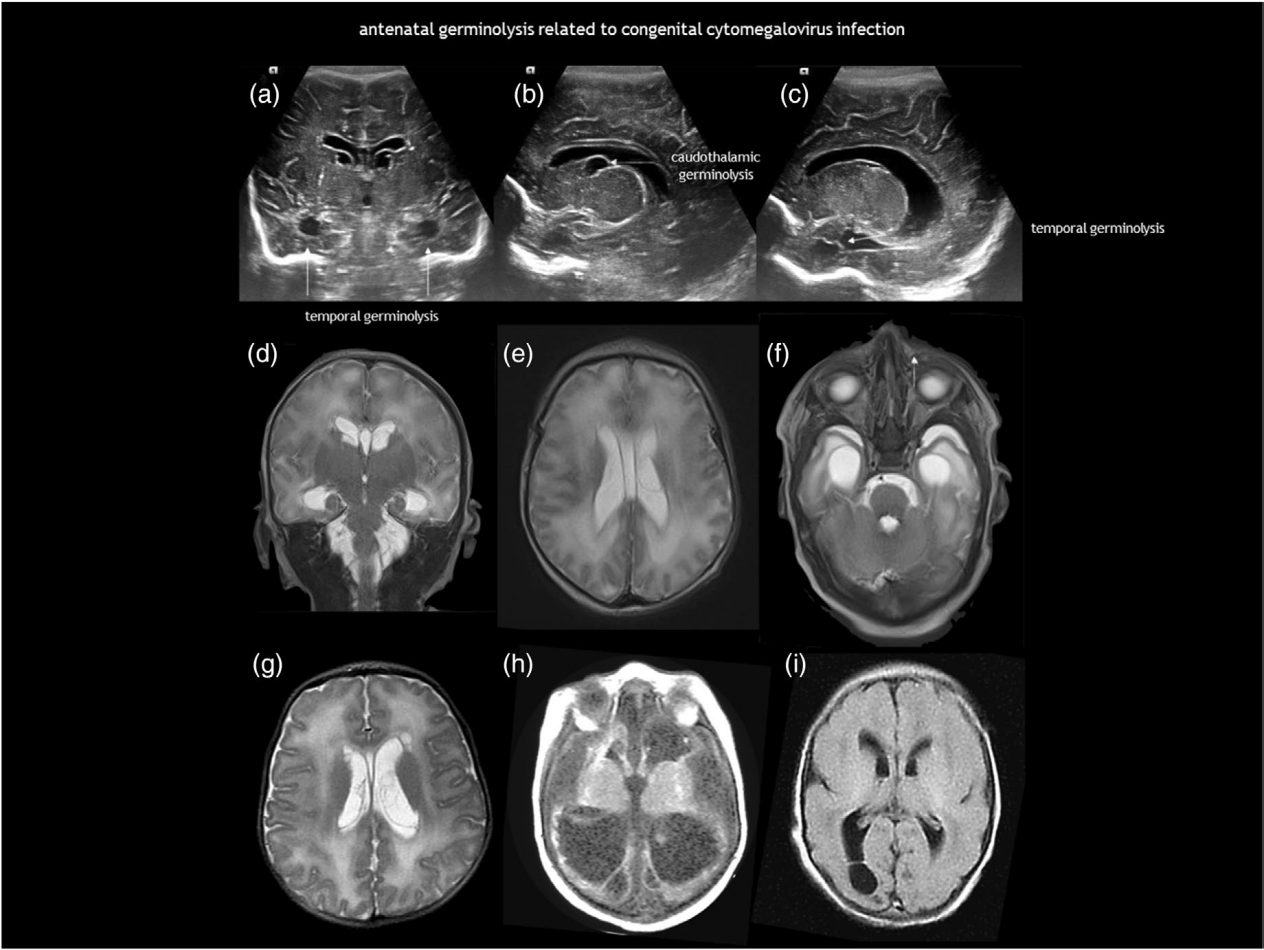
Antenatal germinolysis related to congenital cytomegalovirus (CMV) infection. (a–c) Cranial ultrasonography (CUS) images of an infant with confirmed congenital CMV. (a) Coronal. (b,c) Parasagittal. (d–f) Brain magnetic resonance imaging (MRI) of an infant with confirmed congenital CMV. (d) Coronal T2. (e,f) Axial T2. CUS more clearly reveals caudothalamic germinolysis; however, temporal horn involvement is subtle on CUS and better depicted on MRI. (g) Axial T2 MRI of another infant with congenital CMV showing parafrontal and caudothalamic germinolysis. (h) Coronal T1 MRI of a third infant with congenital CMV, demonstrating severe ventriculomegaly, cortical thinning, smooth cortex, periventricular and central grey nuclei calcifications, and occipital horn septation. (i) Axial T2 fluid‐attenuated inversion recovery MRI of a fourth neonate with congenital CMV, showing septation and adhesion in the right occipital horn.

Germinolysis can also be located in the rostral part of the temporal horns. A similar image of a thin strand crossing the distal end of the ventricle can be seen in the occipital horns. Temporal cysts and occipital horn septations are typical but not specific of congenital cytomegalovirus (CMV) infection (Figure 12). These septations may cause cystic dilatation in utero, which usually becomes less prominent after birth.

Postnatal caudothalamic cystic changes in matrix areas, usually unilateral, typically appear 1 to 2 weeks after germinal matrix haemorrhage is detected in the first days of life. Idiopathic postnatal caudothalamic SEPCs, unrelated to germinal matrix haemorrhage, are commonly observed in infants born preterm in the weeks after birth.^72^ Furthermore, subventricular zone echogenicity, characterized by bilateral, symmetrical, teardrop‐shaped non‐cystic hyperechogenicity within the caudothalamic grooves, can be mistaken for germinal matrix haemorrhage.^73^, ^74^ Subventricular zone echogenicity is associated with motor impairment in infants born preterm with intestinal perforation, probably because of inflammatory injury to the ependymal layer and disruption of the stem cell niche.^75^ Subventricular zone echogenicity is usually a postnatal finding that appears beyond the first week of life, although it can be seen antenatally.

**FIGURE 13:**
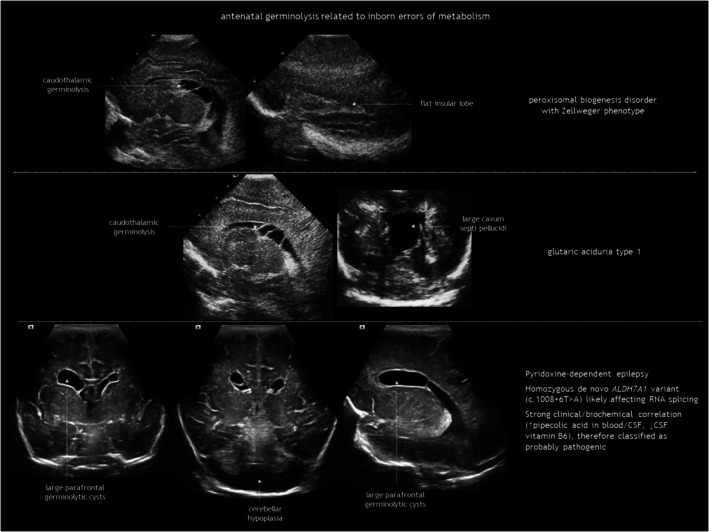
Antenatal germinolysis related to inborn errors of metabolism. Abbreviation: CSF, cerebrospinal fluid.

The possible causes of antenatal germinolysis are summarized in Table 3.^17^, ^18^, ^76^, ^77^, ^78^, ^79^, ^80^, ^81^, ^82^, ^83^, ^84^, ^85^, ^86^, ^87^, ^88^ Small, single caudothalamic SEPCs and parafrontal pseudocysts are generally incidental findings, present in 0.5% to 5% of typically developing neonates.^89^ However, large or multiloculated caudothalamic SEPCs, as well as temporal cysts and occipital horn septations, warrant investigation for underlying disease, particularly CMV and metabolic testing (Table 1, and Figures 12 and 13). Prognosis is good in the absence of associated conditions.^90^ Most SEPCs disappear within the first few months after birth. Shinar et al.^91^ reported benign prefrontal SEPCs in four pairs of sibling foetuses, raising the possibility of familial presentation with dominant pattern of inheritance.

**TABLE 3:** Aetiology of antenatal germinolysis.

Main conditions associated with antenatal subependymal pseudocysts
Idiopathic
Foetal infection: cytomegalovirus, rubella, Zika virus, others^76^, ^77^, ^78^
Antenatal asphyxia^17^, ^18^
Maternal substance abuse (cocaine)^79^
Twin‐to‐twin transfusion syndrome^80^
Congenital heart disease^81^, ^82^, ^83^
Metabolic disorders
Organic acidurias (glutaric aciduria)^84^
Mitochondrial disorders (complex I, IV, pyruvate dehydrogenase deficiency)^85^
Peroxisomal disorders (Zellweger syndrome)^85^
Pyridoxine‐dependent epilepsy^86^
Holocarboxylase synthetase deficiency^87^
Chromosomal abnormalities and genetic disorders^88^

## LENTICULOSTRIATE VASCULOPATHY

**FIGURE 14:**
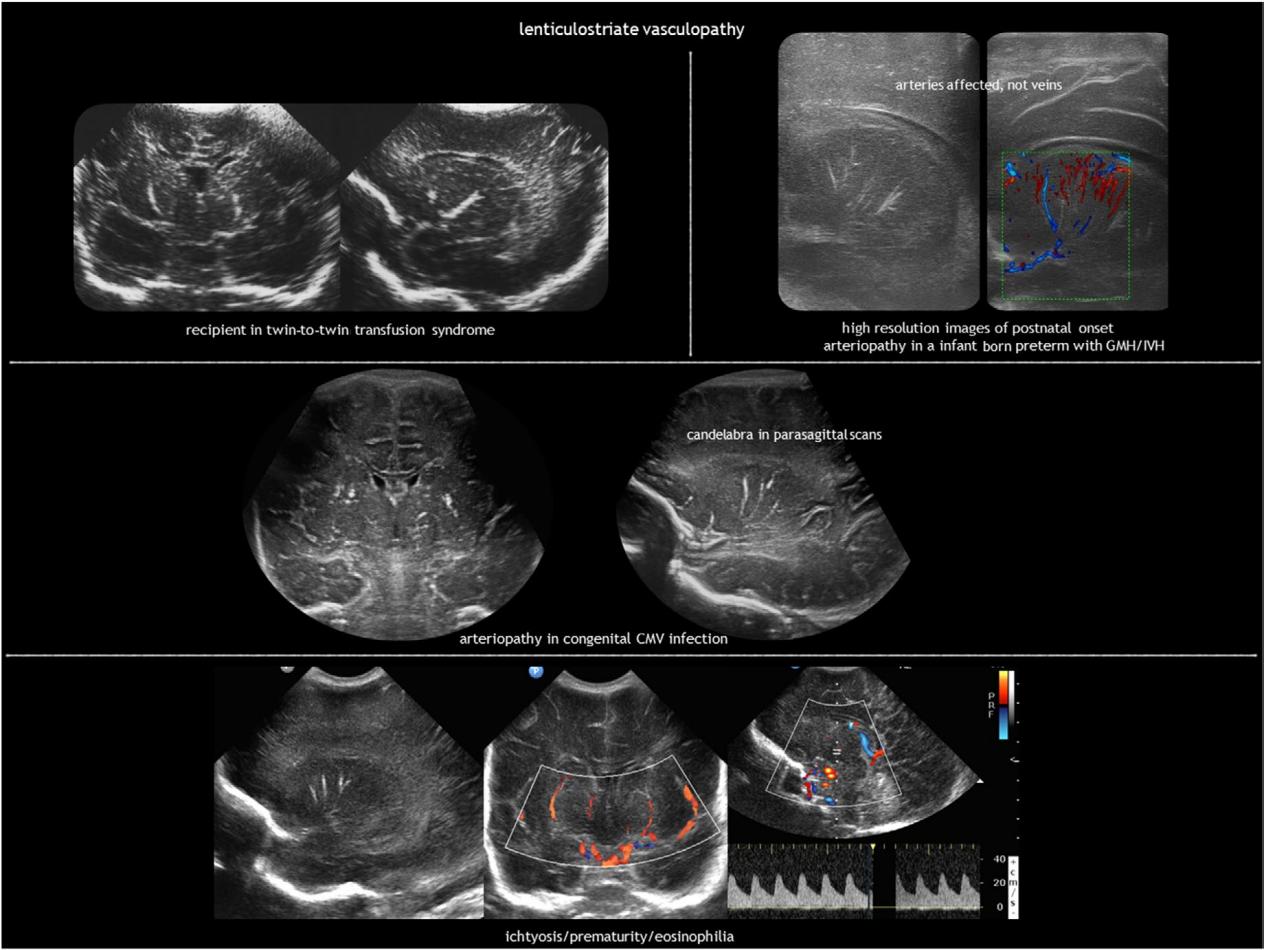
Lenticulostriate vasculopathy. Abbreviations: CMV, cytomegalovirus; GMH, germinal matrix haemorrhage; IVH, intraventricular haemorrhage.

LSV (Figure 14) refers to a characteristic hyperechogenicity of the lenticulostriate arteries.^92^ The pathological correlate consists of inflammation or mineralization of the perforating, medium‐sized arteries supplying the basal ganglia and thalami.^93^, ^94^ LSV is found during routine CUS in 0.4% of all liveborn neonates and in 1.9% to 5.8% of neonates requiring admission because they are preterm or due to other medical reasons.^95^, ^96^, ^97^, ^98^ While not inherently pathological, LSV may serve as a marker for certain conditions (Table 4).^76^, ^85^, ^92^, ^93^, ^94^, ^95^, ^99^, ^100^ Foetal cerebral haemodynamics could have a role in the pathogenesis of idiopathic LSV. Blood flow in the lenticulostriate arteries is high in utero because there is active proliferation of the germinal matrix in the caudothalamic groove.^101^ Minor insults may produce changes in these high‐flow vessels.^95^ Postnatal, late‐onset LSV is associated with IVH^102^ and can also occur after postnatal CMV infection.^103^

**TABLE 4:** Aetiology of antenatal lenticulostriate vasculopathy.

Main conditions associated with antenatal lenticulostriate vasculopathy
Idiopathic
Foetal infection: cytomegalovirus, rubella, Zika virus, herpes simplex virus, syphilis, others^76^, ^92^, ^93^, ^94^, ^95^
Antenatal asphyxia
Foetal alcohol and drug exposure^95^
Neonatal lupus; also cases of antiphospholipid syndrome, alloimmune thrombocytopenia, and maternal idiopathic thrombocytopenic purpura^94^
Twin‐to‐twin transfusion syndrome (recipient)^99^
Congenital heart disease^99^, ^100^
Chromosomal abnormalities: trisomy 21, trisomy 13, 5q deletion, unbalanced chromosome 11 translocation, 46XX/47XXX mosaicism, Miller–Dieker syndrome^95^
Inborn errors of metabolism: Zellweger syndrome, Lowe syndrome, pyruvate dehydrogenase deficiency, desmolase deficiency, glutaric aciduria type 2, infantile arterial calcinosis, storage disorders (e.g. infantile sialidosis)^85^
Syndromes: leukodystrophy, Weaver syndrome, congenital Finnish type nephrosis, Smith–Lemli–Opitz syndrome, linear naevus sebaceus syndrome, contractural arachnodactyly, eosinophilia‐ichthyosis prematurity, incontinentia pigmenti, multisystemic smooth muscle dysfunction caused by *ACTA2* pathogenic variant^95^
Genetic cerebrovascular disease (Table 2)

LSV is often bilateral but asymmetrical. Depending on the angle of insonation and whether the vessel view is transverse or longitudinal, LSV can exhibit a punctate or branching linear ‘candlestick’ pattern, best seen on parasagittal sections. CUS is the neuroimaging modality of choice for depicting LSV; only densely calcified lesions can be seen on CT, gradient echo, or susceptibility‐weighted imaging. LSV can be graded on CUS as minor (one streak), moderate (one or two bilateral streaks), or major (three or more bilateral streaks).^99^ Doppler ultrasound reveals the arterial nature of LSV.

As with SEPCs, prognosis of LSV depends on the cause (Table 1). In the absence of TORCH infections, chromosomal abnormalities, associated congenital anomalies, or other underlying conditions, a typical outcome is expected.

## FOETAL CEREBRAL VASCULAR DISRUPTION IN TWIN PREGNANCIES

Cerebral vascular disruption in foetuses of twin pregnancies primarily results from shared placental circulation in monochorionic twins. Complications such as twin‐to‐twin transfusion syndrome, twin anaemia‐polycythaemia sequence, cord entanglement, or velamentous cord insertion can lead to uneven blood flow, increasing the risk of cerebral ischaemia or stroke. Single intrauterine foetal death in monochorionic twin pregnancies is a serious event that can cause acute anaemia, hypotension, and ischaemia in the surviving twin because of acute twin‐to‐twin transfusion syndrome into the dying twin's low‐pressure vascular system, as well as transchorionic embolism and coagulopathy.

Acquired foetal brain abnormalities in twins have been classified into: (1) brain disruptions (porencephaly, multicystic encephalomalacia, hydranencephaly, and white matter volume loss); (2) malformations of cortical development resulting from disruption in the second trimester (typically polymicrogyria and heterotopia); and (3) sequelae of injury to the cerebellum, including (asymmetrical) cerebellar hypoplasia, cerebellar dysplasia, and Dandy–Walker‐like malformation.^80^, ^104^, ^105^ These abnormalities are presented in Table 5 according to twin type,^80^, ^99^, ^105^, ^106^ with examples illustrated in Figures 8 and 14.

**TABLE 5:** Acquired foetal brain pathology in monochorionic twins.

Type of foetus	Main foetal disruptive brain lesions described in monochorionic twins
Mainly donor	Cystic periventricular leukomalacia^80^, ^105^, ^106^
	Ventriculomegaly^80^, ^105^
	Multicystic encephalomalacia^80^, ^105^
	Hydranencephaly^105^
Mainly recipient	Lenticulostriate vasculopathy^99^
	Intraventricular haemorrhage^80^, ^105^
	Cerebellar haemorrhage (resulting in cerebellar hypoplasia and dysplasia)^80^, ^105^
Either donor or recipient	Germinolysis^80^
	Ischaemic and haemorrhagic stroke^80^, ^105^
	Porencephaly^80^, ^105^
	Schizencephaly^80^, ^105^
	Heterotopia and polymicrogyria^80^, ^105^

Disruptions described outside the brain include renal cortical necrosis, small bowel atresia, gastroschisis, cutis aplasia, and limb amputation.^105^, ^106^

## ANTENATAL BRAIN INJURY IN CONGENITAL HEART DISEASE

Abnormal foetal cerebral circulation can affect brain development in CHD, the most common birth defect. Over the past decades, survival rates have dramatically improved because of advances in surgical techniques and intensive care. While severe neurological sequelae are relatively uncommon in critical CHD, these children are at risk of neurodevelopmental impairments, including mild cognitive deficits, impaired social and communication skills, inattention, and executive function deficits.^107^, ^108^ Multiple factors contribute to these impairments, including genetics, abnormal brain metabolism, and several complications during the preoperative, perioperative, and postoperative periods.^100^, ^108^ Most of these factors converge on CNS dysmaturation and cumulative ischaemia–reperfusion injury. Chronic brain metabolic impairment occurs already during gestation, affecting foetal brain development and disrupting brain growth trajectories in the third trimester.^81^, ^82^, ^109^ Most studies on critical CHD, in particular hypoplastic left heart syndrome and dextro‐transposition of the great arteries, report impaired foetal brain growth.^108^ This altered brain energy metabolism may also increase susceptibility to preoperative and postoperative injuries.

**FIGURE 15:**
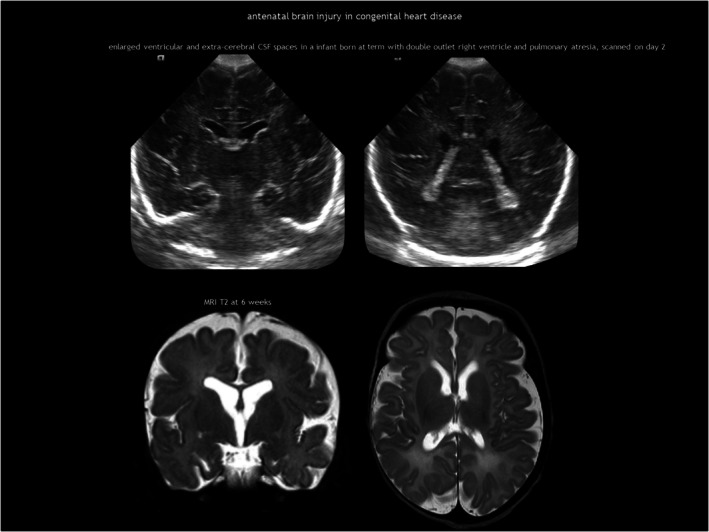
Antenatal brain injury in congenital heart disease. Abbreviations: CSF, cerebrospinal fluid; MRI, magnetic resonance imaging.

White matter damage and arterial ischaemic stroke are the most common types of injury in infants with CHD.^100^, ^108^ While arterial ischaemic stroke does not typically occur in utero, foetal neuroimaging studies have identified white matter abnormalities, such as cysts and signal hyperintensity on T2‐weighted MRI.^81^, ^83^, ^110^ The most common abnormality found in foetal neuroimaging for CHD is enlarged ventricular and extracerebral CSF spaces, a marker of delayed brain development (Figure 15). These qualitative findings correlate with quantitative studies showing impaired brain growth, increased CSF volume, and delayed sulcation.^81^, ^83^, ^109^, ^110^

## DRUG EXPOSURE

Maternal drug use, including medications and recreational drugs, may harm foetal and neonatal health, causing malformations, disruptions, or dysplasias, with effects depending on the dose and timing of exposure.^111^ Several drugs have teratogenic effects on the foetal CNS, leading to structural abnormalities, functional impairments, or neurodevelopmental disorders (Table 6).^79^, ^111^, ^112^, ^113^, ^114^

**TABLE 6:** Main teratogenic drugs affecting the foetal central nervous system.

Teratogen	Potential side effects
Antiseizure medications (carbamazepine, phenytoin, valproic acid)^111^, ^112^	Congenital heart disease, cleft lip or palate, urogenital defects, and neural tube defects
Alcohol^111^, ^113^	Foetal alcohol syndrome consisting of facial dysmorphology (short palpebral fissures, smooth philtrum, thin upper lip), growth restriction, microcephaly, cognitive impairment, behavioural disorders
Cocaine^79^, ^111^	Vascular disruptive‐type abnormalities (low incidence)
Misoprostol^111^	Low incidence of vascular disruptions, including limb reduction defects and Möbius sequence, reported with use to induce abortion
Systemic retinoids, extremely high doses of vitamin A^111^, ^114^	CNS abnormalities (microcephaly, hydrocephalus, intellectual disability), craniofacial defects (micrognathia, cleft palate, ear anomalies), cardiac malformations, thymic hypoplasia, limb abnormalities
Warfarin^111^	Early exposure: nasal hypoplasia, epiphyseal stippling, intrauterine growth restriction. Late exposure: complications of CNS bleeding

Abbreviation: CNS, central nervous system.

## CONGENITAL INFECTIONS AND THE GENETIC DISORDERS THAT MIMIC THEM

### TORCH infections

The acronym ‘TORCH’ refers to a group of congenital infections that share similar clinical characteristics, including a variable combination of intrauterine growth restriction, and involvement of the reticuloendothelial system, CNS, and eye, among other organs. TORCH stands for toxoplasmosis, other agents (classically referring to syphilis), rubella, CMV, and herpes simplex virus. While the acronym is widely accepted for congenital infections overall, an increasing number of agents causing mother‐to‐child transmission, such as parvovirus, varicella zoster virus, and Zika virus, are not included.

The end‐organ effects of TORCH infections are variable, leading to a range of foetal and neonatal clinical manifestations, from asymptomatic to more or less severe symptomatic infections. CNS involvement influences the risk of long‐term neurological sequelae. TORCH infections can have two types of effects on the developing brain. First, the microorganism or the accompanying immune and inflammatory response can lead to tissue destruction. Additionally, if this destruction occurs in the early stages of pregnancy, it can interfere with normal cell organization and differentiation, resulting in dysplastic changes.^1^ Inflammatory and destructive changes include LSV, SEPCs, ventriculomegaly, calcifications, and white matter injury. Typical TORCH‐related brain developmental disorders are malformations of cortical development and cerebellar hypoplasia.^1^, ^76^

CUS and MRI have complementary roles in assessing the CNS of newborn infants with congenital infection. CUS effectively demonstrates LSV, ventriculomegaly, calcifications, and SEPCs. MRI is the criterion standard for evaluating white matter abnormalities, cortical malformations, and the posterior fossa, although the use of the mastoid fontanelle and the transnuchal approach via the foramen magnum facilitate assessment of the cerebellum. While CT has traditionally been the preferred method for detecting intracranial calcification, it is not superior to CUS for this purpose. Furthermore, the health risks associated with ionizing radiation from CT must be considered. Gradient echo and susceptibility‐weighted imaging enhance recognition of brain calcifications with MRI.

Neuroimaging is the best predictor of neurological outcomes in congenital infections. In CMV, the most common TORCH infection, classification systems exist for both foetal and neonatal findings, including both CUS and MRI.^115^, ^116^, ^117^ While normal neuroimaging or mild findings (e.g. LSV, SEPCs, or mild ventriculomegaly) are reassuring, major lesions, such as extensive calcification, brain atrophy, or developmental disorders (e.g. cortical malformation, cerebellar hypoplasia), correlate with poor outcomes. On the other hand, white matter abnormalities pose challenges for treatment and prognosis because of variable neurodevelopmental outcomes.

**FIGURE 16:**
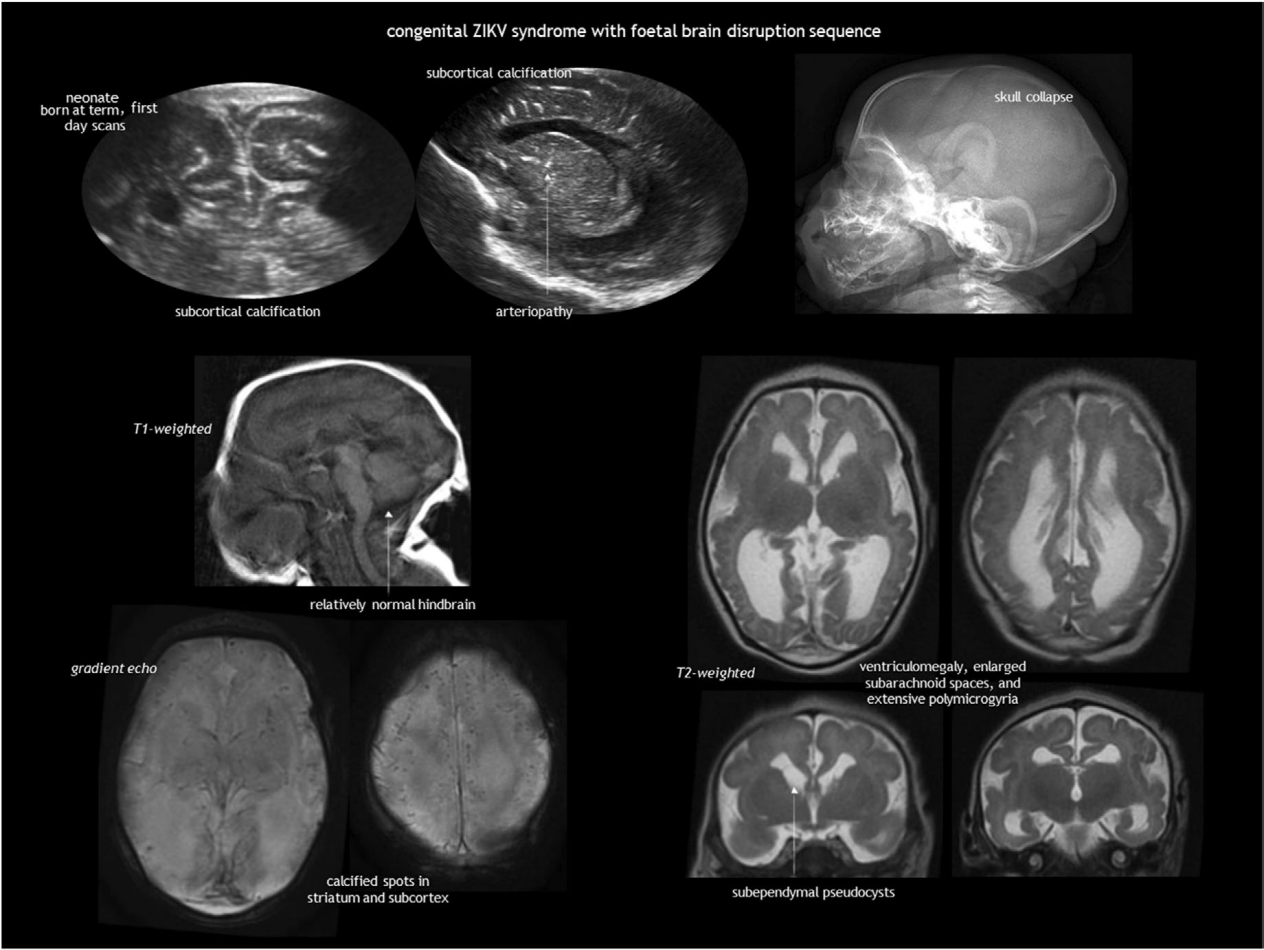
Congenital Zika virus syndrome with foetal brain disruption sequence.

Both infection and vascular disruption can underlie the foetal brain disruption sequence.^118^, ^119^ Features of this sequence are: (1) severe microcephaly; (2) overlapping cranial sutures; (3) prominent occipital bone; and (4) redundant scalp skin. It is hypothesized to result from a loss of brain volume and a decrease in intracranial pressure. This phenotype was rarely reported^120^ before the recent outbreak of Zika virus infection in the Americas. An example of Zika virus‐associated foetal brain disruption sequence^77^, ^78^ is presented in Figure 16.

### Pseudo‐TORCH syndromes

Pseudo‐TORCH syndromes are genetic disorders, mostly autosomal recessive, that exhibit clinical and neuroimaging features similar to TORCH infections but test negative for infectious pathogens.^55^, ^56^, ^121^ These syndromes are characterized by microcephaly, seizures, developmental delay, and intracranial calcification.

**FIGURE 17:**
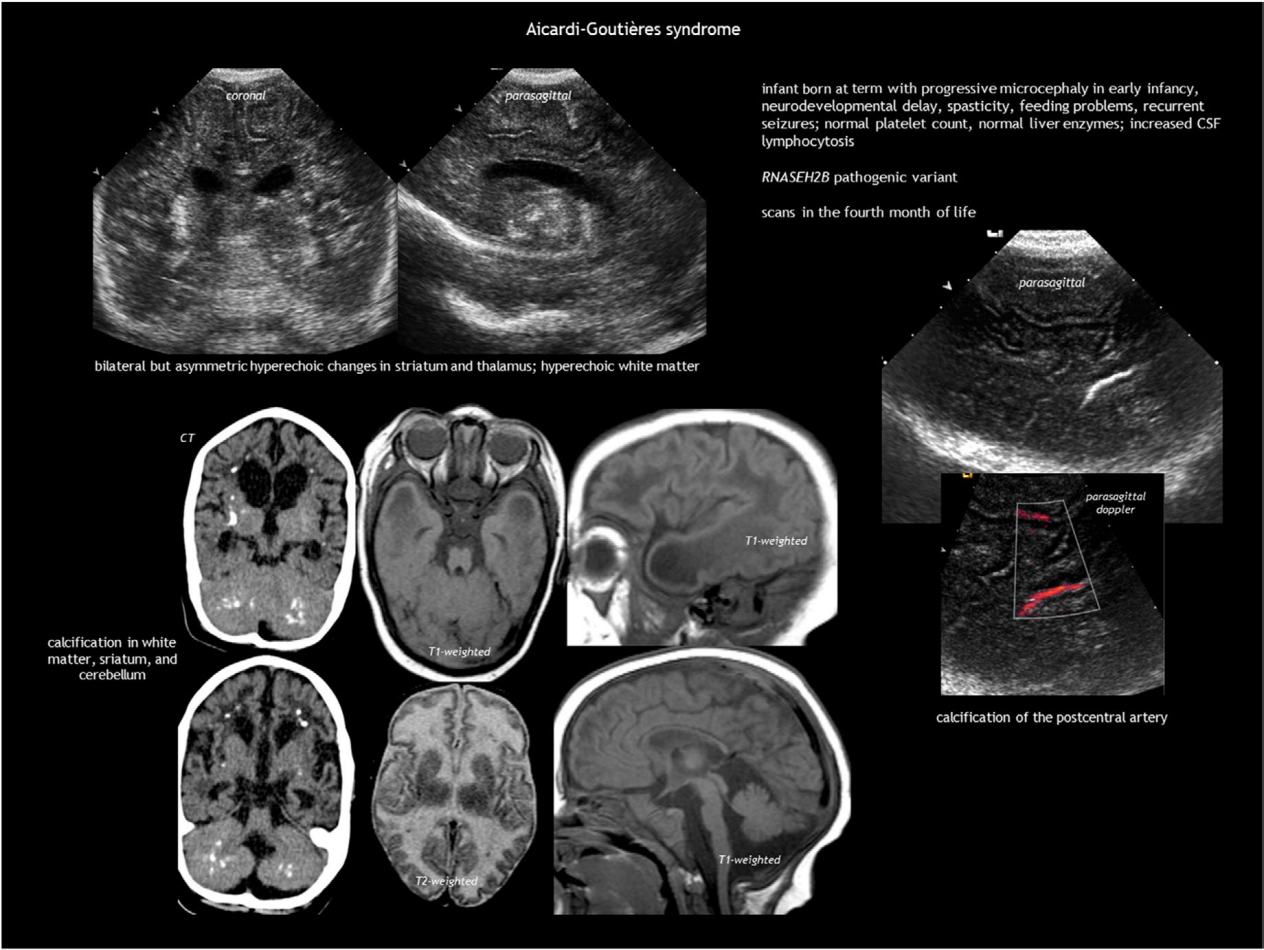
Aicardi–Goutières syndrome. Abbreviations: CSF, cerebrospinal fluid; CT, computed tomography.

Aicardi–Goutières syndrome is a type I interferonopathy that causes early‐onset progressive encephalopathy, which is characterized in its most severe form by cerebral atrophy, microcephaly, leukodystrophy, intracranial calcification, chronic CSF lymphocytosis, and increased interferon alpha in the CSF.^55^, ^56^, ^57^, ^58^ Aicardi–Goutières syndrome is genetically heterogeneous, with severe disease most commonly associated with pathogenic variants in *TREX1*, *RNASEH2C*, and *RNASEH2A*. Aicardi–Goutières syndrome can present as either neonatal or infantile onset. The neonatal form resembles congenital infection, which is characterized by fever, seizures, hepatosplenomegaly, elevated liver enzymes, thrombocytopenia, and anaemia. The more common infantile form follows a period of typical development, leading to subacute severe encephalopathy with irritability, feeding difficulties, and loss of skills. Epilepsy, chilblains, and aseptic febrile episodes are common. Symptoms can last several months before stabilizing, resulting in acquired microcephaly, spasticity, or dystonic posturing as static sequelae. Neuroimaging (Figure 17) shows intracranial calcification (especially in the basal ganglia and white matter), cystic leukodystrophy (predominantly frontotemporal), and cortico‐subcortical atrophy, along with atrophy of the corpus callosum, brainstem, and cerebellum.

Pseudo‐TORCH syndrome‐1 or band‐like calcification with simplified gyration and polymicrogyria results from pathogenic variants in *OCLN*, which encodes occludin, a tight‐junction protein.^55^, ^56^, ^122^ Affected infants have early‐onset seizures, severe microcephaly, multilateral paralysis, bulbar palsy, and severe developmental delay. Neuroimaging findings include rudimentary sulcation with hourglass appearance of the brain, bilateral symmetrical polymicrogyria, and a band of cortical calcification, along with thalamic and brainstem calcification. Additionally, the cerebellum, brainstem, and corpus callosum are hypoplastic.

Pseudo‐TORCH syndrome‐2 is a multisystem disorder caused by pathogenic variants in *USP18*, leading to abnormal activation of the interferon pathway.^13^, ^56^ This severe disease is characterized by microcephaly, liver dysfunction, thrombocytopenia, respiratory failure, and seizures, often resulting in early infant death. Intracranial findings consist of extensive haemorrhagic destruction and calcification, which may develop antenatally or postnatally.

*RNASET2* pathogenic variants cause RNase T2‐deficient cystic leukoencephalopathy, which is characterized by early‐onset severe developmental problems, and a variable combination of microcephaly, seizures, and hearing impairment.^55^, ^56^, ^123^ Neuroimaging resembles congenital CMV, showing white matter abnormalities and anterior temporal cysts. Calcifications may appear in the periventricular area, deep white matter, and basal ganglia.

Pathogenic variants in the tight‐junction protein JAM3 cause severe intrauterine haemorrhagic destruction, accompanied by subependymal calcification, microcephaly, and congenital cataracts. The condition typically results in early death.^12^, ^55^, ^56^

## INBORN ERRORS OF METABOLISM

**FIGURE 18:**
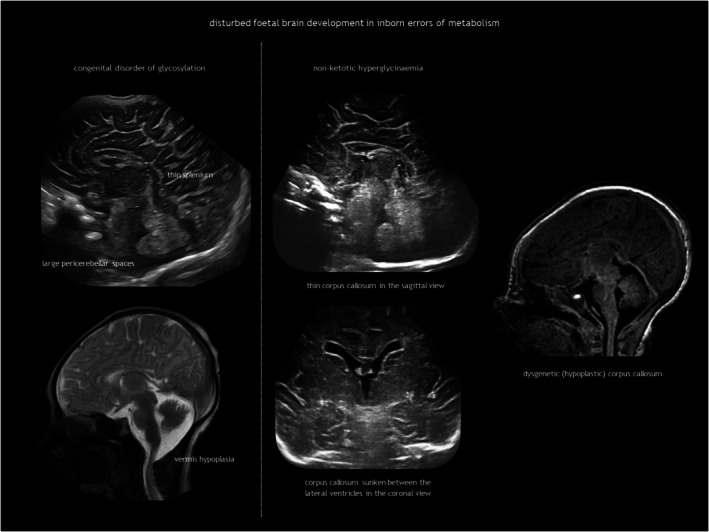
Disturbed foetal brain development in inborn errors of metabolism.

**FIGURE 19:**
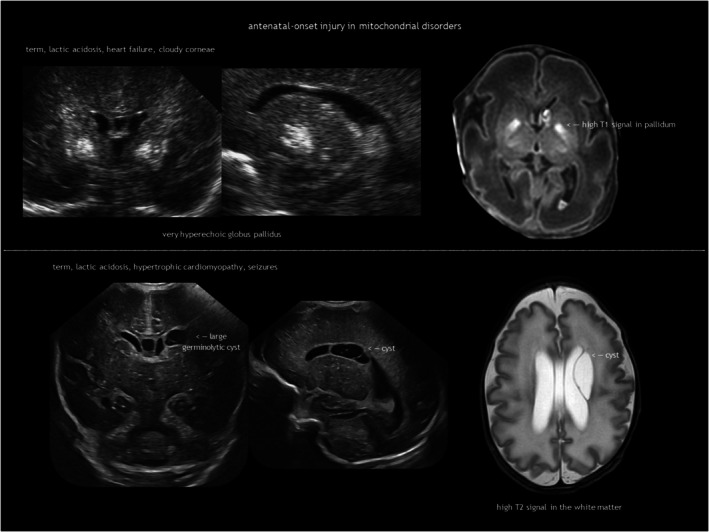
Mitochondrial disorders.

**FIGURE 20:**
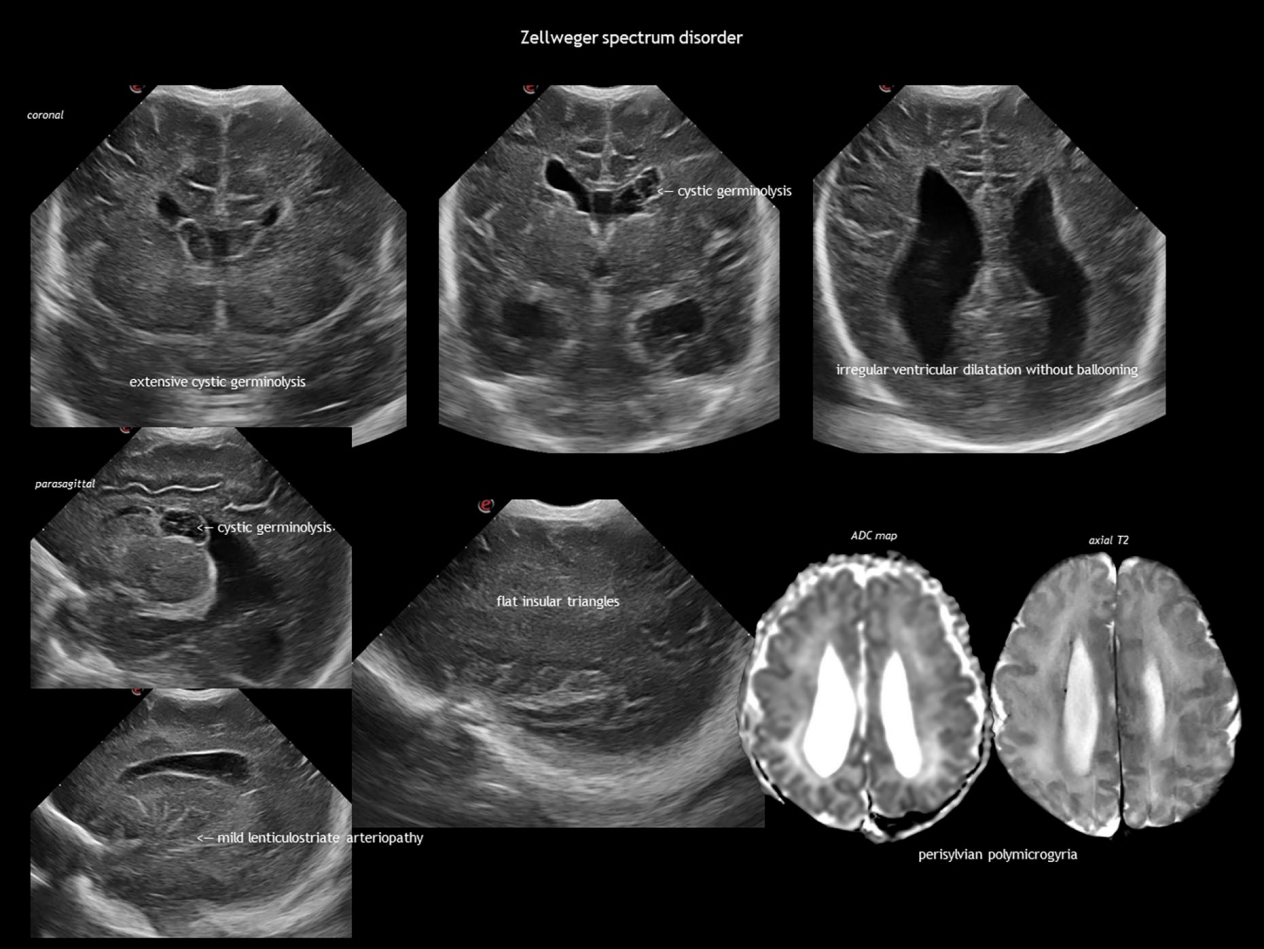
Zellweger spectrum. Abbreviation: ADC, apparent diffusion coefficient.

IEMs result from the deficiency or malfunction of an enzyme or its cofactor, leading to the accumulation or deficit of a specific metabolite. Most of these disorders are autosomal recessive. The association between IEMs and disturbed foetal brain development arises from cellular disruption during critical periods of neurogenesis or migration.^124^, ^125^, ^126^ Prasad et al.^126^ proposed several mechanisms linking IEMs to foetal cerebral dysgenesis, including: (1) neurotoxic metabolite accumulation; (2) defective energy metabolism; (3) disrupted cellular signalling; (4) altered biophysical properties of cell membranes; and (5) organelle dysfunction. Neuroimaging findings in IEMs often consist of a combination of structural abnormalities and acute changes.^85^, ^125^, ^126^ Identifying the distinct features of each disease is helpful in the often difficult diagnostic process. The main IEMs with characteristic foetal brain abnormalities are described in Table 7,^66^, ^84^, ^85^, ^124^, ^125^, ^126^ with examples shown in Figures 13, 18, 19 and 20.

**TABLE 7:** Main inborn errors of metabolism associated with foetal brain pathology.

Inborn error of metabolism according to category		Biochemistry and genetics	Clinical manifestations	Neuroimaging features
Aminoacidopathies	Maternal phenylketonuria^124^, ^125^, ^126^	Elevated phenylalanine in the mother Phenylalanine hydroxylase deficiency, AR	Microcephaly, developmental delay, congenital heart disease, intrauterine growth restriction	Dysgenesis of the corpus callosum, delayed myelination
	Non‐ketotic hyperglycinaemia^85^, ^124^, ^125^, ^126^	Elevated glycine in plasma, urine, and CSF. Abnormally high CSF and plasma glycine ratio Deficiency in glycine cleavage system enzymes (P, H, T, or L proteins), AR	Lethargy, hypotonia, myoclonic seizures, apnoea	Agenesis or thinning of the corpus callosum, demyelination, gyral abnormalities
Organic acidaemias	Glutaric aciduria type 1^84^, ^125^, ^126^	Elevated glutaric acid and 3‐hydroxyglutaric acid Glutaryl‐coenzyme A dehydrogenase deficiency, AR	Macrocephaly, dystonia	Macrocrania, frontotemporal atrophy, incomplete opercularization, SEPCs, striatal atrophy
Mitochondrial disorders	Disorders of the respiratory chain^66^, ^85^, ^124^, ^125^, ^126^	Primary lactic acidosis Pathogenic variants in mitochondrial DNA (mtDNA) or nuclear DNA (nDNA), affecting components of the electron transport chain complexes I–V. Inheritance can be maternal (mtDNA), autosomal recessive (nDNA), or X‐linked	Hypotonia, lethargy, poor sucking, encephalopathy, seizures. Cardiomyopathy and other systemic involvement	Bilateral symmetric lesions in the deep grey nuclei, cortical and cerebellar atrophy, white matter abnormalities
Pyruvate metabolism	Pyruvate dehydrogenase deficiency^85^, ^124^, ^125^, ^126^	Primary lactic acidosis X‐linked‐dominant (PDHA1, most common); AR (PDHB, DLAT, DLD, PDHX)	Encephalopathy	Cerebral atrophy, cystic encephalomalacia, basal ganglia lesions, callosal dysgenesis
Cholesterol biosynthesis disorders	Smith–Lemli–Opitz syndrome^124^, ^125^, ^126^	Low cholesterol, elevated 7‐dehydrocholesterol Deficiency of 7‐dehydrocholesterol reductase, AR	Microcephaly, facial anomalies, syndactyly (2–3 toe fusion), urogenital anomalies	Holoprosencephaly (in severe cases), hypoplasia/agenesis of the corpus callosum, cerebellar hypoplasia
Peroxisomal disorders	Peroxisome biogenesis disorders or single peroxisomal enzyme disorders^85^, ^125^, ^126^	Elevated plasma very‐long‐chain fatty acids, bile acids, phytanic, pristanic, and pipecolic acids; low plasmalogen levels Variants in over 25 *PEX* genes, mostly AR	Classified into: Cerebrohepatorenal syndromes (including Zellweger spectrum): liver, kidney, and neurological abnormalities. Marked hypotonia, hyporeflexia, seizures. Craniofacial dysmorphism (prominent forehead, large fontanelle, hypoplastic supraorbital ridges, upslanted eyes, epicanthal folds, broad nasal bridge, high‐arched palate, abnormal earlobes) Rhizomelic chondrodysplasias: rhizomelia, developmental delay, cataracts, and joint contractures Refsum disease: progressive vision loss and neuropathy, typically presenting in adolescence X‐linked adrenoleukodystrophy	Cortical malformation (typically frontal and perisylvian polymicrogyria), hypomyelination, SEPCs
Glycoprotein metabolism	Congenital disorders of glycosylation^85^, ^125^, ^126^	More than 10 subtypes, CDG type 1a (PMM2‐CDG) is the most common Pathogenic variants in genes involved in glycosylation (*PMM2* gene for CDG1a), AR	Dysmorphic features (facial anomalies, inverted nipples), hypotonia, multisystem involvement (liver dysfunction, coagulation disorders, endocrine abnormalities)	Cerebellar hypoplasia

Abbreviations: AR, autosomal recessive; CDG, congenital disorder of glycosylation; CSF, cerebrospinal fluid; DLAT, dihydrolipoyllysine‐residue acetyltransferase component of pyruvate dehydrogenase complex, mitochondrial; DLD, dihydrolipoamide dehydrogenase; PDHA1, pyruvate dehydrogenase E1 component subunit alpha, somatic form, mitochondrial; PDHB, pyruvate dehydrogenase E1 component subunit beta, mitochondrial; PDHX, pyruvate dehydrogenase protein X component, mitochondrial; PMM2, phosphomannomutase 2; SEPC, subependymal pseudocyst.

## CONCLUSION

The foetal brain is vulnerable to teratogens, intrauterine infection, hypoxia‐ischaemia, haemorrhage, and trauma. Foetal brain circulation is characterized by a highly vascularized germinal matrix, making the foetus and infant born preterm prone to germinal matrix haemorrhage. However, germinolysis and LSV are more common. Foetal brain injury often leads to cavitation, with the pattern varying depending on the pathophysiology and timing. Porencephaly and schizencephaly are focal disruptive defects. Congenital multicystic encephalomalacia results from prolonged, partial hypoxic‐ischaemic injury in the third trimester, while hydranencephaly arises from very early severe occlusion of the internal carotid arteries.

Monochorionic twins are at risk of antenatal vascular disruptive lesions, mostly because of complications from placental anastomoses. Major disruptive brain abnormalities are rare in CHD; nevertheless, impaired foetal brain growth and development is rather common in critical CHD.

Maternal use of medications or recreational drugs can harm the foetal CNS, causing malformations, disruptions, or dysplasias, depending on the drug, dose, and timing of exposure. Congenital infections are also potentially teratogenic, producing characteristic combinations of inflammatory and destructive effects and developmental disruptions in the foetal brain. Some genetic disorders may mimic these patterns and should be taken into consideration in the absence of evidence of TORCH infection. Lastly, IEMs can disrupt foetal brain development, with specific patterns of neuroimaging abnormalities that can help guide diagnosis.

With increased genetic testing, a growing number of genetic conditions predisposing to foetal stroke or other disruptive brain abnormalities are likely to be identified. Both neuroimaging phenotype recognition and genetic testing aid aetiological diagnosis, which is the basis for potential individualized treatment, more precise counselling, and primary and secondary prevention. As the ability to identify genetic causes improves, it is crucial to continuously review the pathophysiology and aetiology on a case‐by‐case basis. Recognizing patterns suggestive of either genetic or non‐genetic origins will facilitate better parental counselling regarding recurrence risks, particularly in cases where the results of genetic testing are normal.

## CONFLICT OF INTEREST STATEMENT

The authors have stated that they had no interests which might be perceived as posing a conflict or bias.

## Data Availability

Not applicable (review content).
